# Development of 11- to 16-year-olds’ short-term power output determined using both treadmill running and cycle ergometry

**DOI:** 10.1007/s00421-019-04146-1

**Published:** 2019-04-26

**Authors:** Neil Armstrong, Jo Welsman, Saul Bloxham

**Affiliations:** 1 Children’s Health and Exercise Research Centre, University of Exeter, St Lukes Campus, Exeter, EX1 2LU UK; 2 School of Sport, Health and Well Being, Plymouth Marjon University, Derriford Road, Plymouth, PL6 8BH UK

**Keywords:** Mean power, Multilevel modelling, Non-motorized treadmill test, Peak power, Wingate anaerobic test, Youth

## Abstract

**Purpose:**

To investigate the development of peak power output (PP) and mean power output (MP) during two different modes of exercise in relation to sex and concurrent changes in age, body mass, fat-free mass (FFM), maturity status and, in the case of MP, peak oxygen uptake ($\dot{V}{\text{O}}_{2}$).

**Methods:**

PP and MP were determined cycling against a fixed braking force (Wingate anaerobic test) and running on a non-motorized treadmill. Peak $\dot{V}{\text{O}}_{2}$ was determined using cycle ergometry and treadmill running. 135 (63 girls) students initially aged 11–14 years were tested over 2 days on three annual occasions. The data were analysed using multiplicative allometric modelling which enables the effects of variables to be partitioned concurrently within an allometric framework. Multiplicative models were founded on 301 (138 from girls) determinations of PP and MP on each ergometer.

**Results:**

With body mass controlled for, both PP and MP increased with age but maturity status did not independently contribute to any of the multiplicative allometric models. Boys’ PP and MP were significantly (*p* < 0.05) higher than girls’ values on both ergometers. On both ergometers in both sexes, the most powerful morphological influence on PP and MP was FFM. Ergometer-specific peak $\dot{V}{\text{O}}_{2}$ had a significant (*p* < 0.05), additional effect in explaining the development of MP.

**Conclusions:**

The development of short-term power output is sex specific but within sex multiplicative allometric models of running- and cycling-determined PP and MP were similar, suggesting that either mode of exercise can be used in future studies of short-term power output in youth.

## Introduction

Young people’s aerobic power (peak oxygen uptake; peak $\dot{V}{\text{O}}_{2}$) determined running on a treadmill or pedalling on a cycle ergometer is well documented but short-term power output (STP) principally reliant on anaerobic metabolism is less extensively researched. Ethical and technological constraints restrict direct measurement of the intramuscular rate of energy production and current knowledge of the development of STP is founded on cross-sectional analyses of performance outcomes. Paediatric research has primarily focused on the assessment of STP during cycling and unlike research into the development of aerobic power little attention has been given to its determination during running.

Although several methods of assessing STP have been explored, understanding of STP in childhood and adolescence is principally derived from performance on variants of the Wingate anaerobic test (WAnT) (Williams and Ratel 2017). Power output in the WAnT is calculated from maximal pedalling cadence against a fixed braking force with peak power output (PP) recorded within a few seconds of exercise onset and total power output averaged over the 30 s test period and expressed as mean power output (MP). Since its introduction by Cumming (1973) and subsequent development at the Wingate Institute, the WAnT has been shown to be a robust and reliable test (Inbar et al. 1996). Paediatric physiologists have, however, developed a plethora of different WAnT protocols including the use of pre-test warm-ups (Inbar and Bar-Or 1975), rolling starts (Armstrong et al. 1997), and toe clips (Lavoie et al. 1984). The time (e.g. 1 s, 3 s, or 5 s) over which PP is recorded varies across studies (Williams and Ratel 2017) and some researchers have incorporated estimations of the work done to overcome the inertia of the flywheel and the internal resistance of the cycle ergometer (Chia et al. 1997) into their calculations. With children and adolescents, concerns have been raised about the ideal body mass-related braking force (Bar-Or 1993). Some researchers have identified individual optimal braking forces and, therefore, optimized peak power (OPP) through a series of short sprints performed against a range of randomly introduced braking forces. Across the 11- to 16-year-old age group, PP has been demonstrated to be generally resilient to moderate variations around the conventional braking force of 0.74 N kg^−1^ (Dotan and Bar-Or 1983; Santos et al. 2002), but if the braking force is set to optimize PP it may be too high for optimal MP determination. As the WAnT progresses, increasing fatigue causes a reduction in pedal cadence, thus affecting the power-to-velocity ratio resulting in a fall in power output and a lower MP. Moreover, MP is a function of interplay between anaerobic and aerobic metabolism with both girls and boys reaching ~ 65–75% of their peak $\dot{V}{\text{O}}_{2}$ in a WAnT (Chia 2006). The influence of peak $\dot{V}{\text{O}}_{2}$ on the development of 11- to 16-year-olds’ MP is unknown.

Cross-sectional studies of WAnT-determined PP and MP are plentiful but the use of a variety of methodologies and power output calculations has precluded confident comparisons of data across studies. Prior to the present project, there were no longitudinal studies of the PP and MP of both boys and girls and only two short duration longitudinal studies with small numbers of boys (Duché et al. 1993; Falk and Bar-Or 1993). There are large differences in reports of ‘typical’ age-related absolute values (i.e. in W) of PP and particularly MP which includes significant but, as yet unquantified, contributions from $\dot{V}{\text{O}}_{2}$. Nevertheless, data trends with age, at least for PP, are consistent and indicate that it increases with age until ~ 13 years with no significant sex difference. From ~ 13 to 14 years of age, the extant data indicate that boys’ PP markedly increases into young adulthood whereas girls experience a smaller increase with age over the same time period (Van Praagh and Doré 2002).

Two large, cross-sectional studies of OPP used an allometric model to investigate the relative contribution of morphological variables to the total variance in OPP. OPP was reported to be correlated with body mass but more strongly related to fat-free mass (FFM) in both sexes (Doré et al. 2000, 2001). There is a paucity of evidence to confirm these relationships as both cross-sectional and longitudinal studies have generally ‘corrected’ for morphological variables by simply dividing PP and MP with either body mass or FFM and expressing them as W kg^−1^ (i.e. ratio scaling). It has been shown theoretically and empirically (Armstrong and Welsman 2019a; Tanner 1949; Welsman and Armstrong 2019) that ratio scaling has neither a sound scientific rationale nor statistical justification. Widespread and erroneous use of this scaling technique has clouded the interpretation of developmental exercise physiology and these analyses have confounded rather than clarified our understanding of the development of STP.

Running-related activities and sports are far more common in youth than cycling and, having different muscle recruitment and activation patterns, WAnT-determined PP and MP are unlikely to provide a valid assessment of STP during running-related activities. This is reflected by the low-to-moderate correlations (*r* = ~ 0.5–0.7) between WAnT external power indices and sport-related performances such as sprint running (Almuzaini 2000; Bencke et al. 2002; Hoffman et al. 2000).

The need for a laboratory running test of STP has been recognized since Margaria et al.'s (1966) seminal report of staircase running. Lakomy’s (1984, 1987) studies of elite adult athletes appear to have been the first to use a non-motorized treadmill (NMT) but it was Van Praagh et al. (Fargeas et al. 1993; Van Praagh et al. 1993) who initially explored the performance of elite youth athletes running on an NMT. They used 10 s sprints and published their research in abstract form only before stopping further development due to child safety concerns. Falk et al. (1996) explored the use of an NMT test (NMTT) to assess the PP of youth athletes over the number of ‘complete stride cycles nearest to 2.5 s’ and MP over 30 s. They observed that many participants had to quit earlier than 30 s or their performance became very irregular and deteriorated badly during the final seconds. They reported performance over 20 s rather than 30 s and do not appear to have persisted with development of an NMTT.

Sutton et al. (2000) developed a laboratory-based NMTT of STP suitable for longitudinal analyses of 8- to 18-year olds. The testing station was developed from Lakomy’s (1987) prototype with a strong emphasis on safety and a built-in adjustable safety harness. The safety harness was rarely used even with young children (on ~ 5% of occasions with 8-year olds) but enhanced the confidence of both children and adolescents who following habituation to the test were able to produce maximal performances over a full 30 s period (i.e. to mirror WAnT MP performance). On completion of the testing station, no methodological or safety problems were encountered in reliability studies and the typical error of NMTT-determined values 1 week apart was reported as 6% for PP and 5% for MP which compares very favourably with the test–retest reliability of the WAnT (Williams and Ratel 2017). The NMT testing station has subsequently been regularly and successfully used with 11- to 16-year olds to investigate, for example, repeated sprint ability (Oliver et al. 2006), recovery profiles following maximal cycling and running (Ratel et al. 2004), age-related recovery profiles (Ratel et al. 2006), and simulated sport-related performances (Oliver et al. 2007). The establishment of an NMTT to complement the WAnT enables exercise mode-specific (i.e. running or cycling) comparisons of the development of STP and peak $\dot{V}{\text{O}}_{2}$.

Collectively studies which have focused on PP and MP in relation to age or a single morphological variable at specific ‘snapshot’ moments in time have provided limited insights into the development of STP. But the emergence (Aitkin et al. 1981) and regular refinement (Rasbash et al. 2018) of multilevel regression modelling has opened up new avenues of research in developmental exercise physiology. Multilevel allometric modelling enables the effects of variables such as age, body mass, FFM, and maturity status to be partitioned concurrently within an allometric framework to provide a flexible interpretation of the development of exercise performance variables.

Nevill et al. (1998) introduced multiplicative, allometric modelling to paediatric sport science with a re-analysis of previously published data from elite youth athletes and simultaneously with the present authors applied it to a prospective study of the development of 11- to 13-year olds’ peak $\dot{V}{\text{O}}_{2}$ (Armstrong et al. 1999). There have been few subsequent publications of longitudinal data and applications of multiplicative allometric modelling to developmental exercise physiology have been remarkably sparse. There were no published longitudinal studies of the PP and MP of 11- to 16-year-old girls and boys before the present project and no studies had investigated the sex-specific influence of concurrent changes in age, maturity status, body mass, and FFM on the development of either STP or peak $\dot{V}{\text{O}}_{2}$. The contribution of peak $\dot{V}{\text{O}}_{2}$ to the development of MP assessed running or cycling had not been explored.

The primary purposes of the present study were, therefore, to enhance understanding of the development of STP in youth by (1) adopting a multiplicative, allometric modelling approach to investigate the sex-specific development of PP and MP from 11 to 16 years of age in relation to concurrent changes in age, maturity status, body mass, and FFM and, in the case of MP, peak $\dot{V}{\text{O}}_{2}$; and (2) comparing the development of PP and MP from 11 to 16 years of age as assessed by maximal intensity running and cycling exercise. A subsidiary purpose was to compare and contrast the development of STP and peak $\dot{V}{\text{O}}_{2}$ in the same young people from 11 to 16 years.

## Methods

### Participants

135 (63 girls) 11- to 14-year-old students volunteered to participate in a project involving longitudinal studies of aerobic and anaerobic fitness. The project received ethical approval from the District Health Authority Ethical Committee and all participants and their guardians provided written informed consent to participate. The present paper is the fourth of the final reports from the project (Armstrong and Welsman 2019a, b, c). The cycle ergometer-determined data have been integrated into a review of 10- to 18-year-olds’ STP published elsewhere (Armstrong and Welsman 2019b) but neither of the present data sets have previously been reported.

### Experimental procedures

Prior to data collection, the students were well habituated to the laboratory environment, to the laboratory personnel who were unchanged during the study, and to the experimental procedures. Students visited the laboratory to familiarize themselves with walking, jogging, and sprinting maximally on an NMT and pedalling maximally against an appropriate fixed braking force on a cycle ergometer. All participants in the project acquired the skills necessary to confidently sprint maximally on the NMT prior to testing sessions. The habituation sessions were followed by 2 days of testing on three annual occasions. The annual tests took place in the same week of the year in randomized order over 2 days with a minimum 2 h rest interval between tests. Individual ages were computed from date of birth at each test session.

#### Anthropometry

Anthropometric measures were taken as described by the International Biological Programme (Weiner and Lourie 1981) and apparatus was calibrated according to the manufacturers’ instructions. Body mass was determined using Avery balance scales (Avery, Birmingham, UK). Skinfold thicknesses over the triceps and subscapular regions were measured using Holtain skinfold callipers (Holtain, Crmych, Dyfed, UK). Maturity status was visually assessed by the Research Centre nurse using the indices for pubic hair (PH) development described by Tanner (1962). FFM was estimated from skinfolds, body mass, and maturity status using youth-specific equations (Slaughter et al. 1988).

#### Wingate anaerobic test

All tests were conducted on the same friction-loaded cycle ergometer (Monark 814E, Monark-Crescent AB, Varberg, Sweden) interfaced with a microcomputer which was calibrated according to the manufacturer’s instructions. The seat height and handlebars were adjusted for each participant and, in accord with the extant literature, the braking force was set in relation to body mass at 0.74 N kg^−1^ (Williams and Ratel 2017).

Following a standardized 3 min warm-up involving pedalling at 60 rev min^−1^ interspersed with three all-out sprints lasting 2–3 s, the WAnT commenced from a rolling start pedalling with toe clips fixed to the pedals at 60 rev min^−1^ against a minimal resistance (i.e. with weight basket supported). When a constant pedal rate was attained, a countdown of ‘3-2-1-Go’ was given, the braking force was applied, and the computer activated. On the signal ‘Go’ participants, with strong verbal encouragement, cycled as fast as possible and power output, corrected for inertia and load as described by Chia et al. (1997), was calculated each second for the 30 s duration of the test. The highest power output achieved in 1 s was recorded as PP and MP was calculated as the average power output over the 30 s duration of the test.

#### Non-motorized treadmill test

The NMTT station was a permanent, purpose-built fixture incorporating a level Woodway TRAMP NMT (Woodway BmbH, Weil am Rhein, Germany) and safety frame bolted to the laboratory floor. PP and MP were determined running on the NMT modified to incorporate an electronic sensor to monitor belt speed. To measure horizontal forces, a strain gauge (Novatech Measurements, St Leonards–on-Sea, UK) fixed to a wall bracket adjustable to the size of the individual student was connected to participants via an extensible tether. The strain gauge was re-calibrated before each test. The output forces from the force sensor and speed sensor were connected to a multifunction interface card installed on a computer for data processing. A visual display provided feedback for the initiation of the test. An external 750 W motor attached to the NMT revolved the belt at a constant speed of 19 km h^−1^ (5.28 m s^−1^) and was run for 5 min prior to each test to standardize the internal resistance of the belt and disengaged during testing. Test development, detailed specifications of the testing station and studies establishing the safety, feasibility and reliability of the NMTT with children as young as 8 years of age have been published elsewhere (Sutton et al. 2000).

Following a standardized low intensity, 3 min warm-up including a 2–3 s sprint, each participant jogged up to a speed of 10 km h^−1^ (2.78 m s^−1^) while viewing a visual display. Once this speed was attained, a countdown of ‘3-2-1-Go’ was given and the data software activated simultaneously. On the signal ‘Go’ participants, with strong verbal encouragement, sprinted as fast as possible for 30 s and power output was calculated as the product of horizontal force and NMT belt speed, over 1 s intervals. Experience over 20 years of using an NMT has shown that some participants lean forcibly into the belt yielding an inflated value of PP within the first 2 s. To avoid this, the first 2 s of data were erased from the analysis and the highest 1 s value achieved thereafter recorded as PP. MP was calculated as the average power output over the last 28 s of the test.

#### Determination of peak oxygen uptake

In a parallel component of the project, the students’ peak $\dot{V}{\text{O}}_{2}$ was determined annually on both a cycle ergometer and a treadmill within 24 h of the WAnT- and NMTT- determined PPs and MPs described herein. Methodological details, descriptive data, and multilevel models incorporating age, body mass, skinfold thicknesses, estimated FFM, and maturity status have been reported elsewhere (Armstrong and Welsman 2019c). In summary, depending on age, treadmill tests began at a belt speed of 7 km h^−1^ (1.94 m s^−1^) or 8 km h^−1^ (2.22 m s^−1^) and increased by 1 km h^−1^ (0.28 m s^−1^) every 2 min until a speed of 10 km h^−1^ (2.78 m s^−1^) was reached. Subsequently, belt speed was held constant and the gradient was incrementally increased by 2.5% every 2 min until voluntary exhaustion. Cycle ergometer tests commenced at an exercise intensity of either 60 or 80 W depending on age. The pedal cadence was fixed at 60 rpm and the exercise intensity increased by 20 W every 2 min until voluntary exhaustion. The highest 30 s $\dot{V}{\text{O}}_{2}$ attained was accepted as a maximal index if clear signs of intense exertion (e.g. hyperpnea, facial flushing unsteady gait, profuse sweating) were demonstrated and supported by a respiratory exchange ratio greater than 1.00 and a heart rate which was levelling-off over the final stages of the test at a value within 5% of the mean ergometer-specific maximal heart rates we have previously reported for 11- to 16-year-olds (Armstrong et al. 1991). All participants reported herein satisfied these criteria on all test occasions.

### Data analyses

Data were analysed using SPSS v25 (IBM SPSS Statistics). To describe age, body mass, and estimated FFM, relationships with PP and MP data were graphed by ergometer and sex and Pearson product moment correlation coefficients were computed.

Factors associated with the development of PP and MP were analysed using multilevel regression modelling (MLWin v3.02, Centre for Multilevel Modelling, University of Bristol, UK). The multiplicative, allometric approach introduced by Nevill et al. (1998) was adopted (Eq. 1).

1 $$ y = {\text{body mass}}^{k} \times { \exp }(a_{j} + b \times {\text{age}} + c \times {\text{age}}^{ 2} )\varepsilon_{ij} . $$

Log transformation linearizes the model as in Eq. (1) forming the starting point for analyses:

2 $$ { \log }_{\text{e}} y = k \times { \log }_{\text{e}} \,{\text{body mass}} + a_{j} + b \times {\text{age}} + c \times {\text{age}}^{ 2} + { \log }_{\text{e}} (\varepsilon_{ij} ). $$

All parameters were fixed with the exception of the constant (*a*) which was allowed to vary randomly at level 2 (between individuals) and the multiplicative error ratio (*ε*) which also varied randomly at level 1 (within individual) as denoted by the subscripts *i* (level 1 variation) and *j* (level 2 variation). Age was centred on the group mean. From the baseline model of age, age^2^, and body mass, additional explanatory variables were explored. In the initial models, sex differences were investigated using the indicator variable boys = 0, girls = 1 which sets the boys’ constant as the baseline from which the girls’ parameter is allowed to deviate. The interaction term age by sex investigates differential development of PP and MP between girls and boys and the age^2^ term indicates changes in the size of the age effect as the rate of change in growth decreases. In the sex-specific analyses, from the baseline model of age, age^2^, and body mass (or estimated FFM in place of body mass), additional explanatory variables including indicator variables for maturity status (i.e. PH stages 2, 3, 4, and 5) which set stage 1 as the baseline from which effects due to maturation can be explored. In the age and body mass (but not estimated FFM) models, sum of triceps and subscapular skinfold thicknesses (as log_e_ sum of skinfolds) was also entered. Finally, cycle ergometer-determined peak $\dot{V}{\text{O}}_{2}$ was entered into the WAnT-determined MP, sex-specific models of age, age^2^, body mass, and skinfold thicknesses. Treadmill-determined peak $\dot{V}{\text{O}}_{2}$ was entered into the NMTT-determined MP, sex-specific models of age, age^2^, and estimated FFM.

Parameter estimates were considered significant (*p* < 0.05) where their value exceeded 2 × the standard error (SE). The change in deviance statistic (− 2 × log-likelihood) was used to assess the goodness of fit of the models. A comparison of the goodness of fit of the different models was obtained from the change in the deviance statistic (− 2 × log-likelihood) with reference to the number of fitted parameters. In a comparison of two models with the same number of fitted parameters, the model with the smallest − 2 × log-likelihood reflects that with the best fit. Additional parameters contribute to improved fit from the change in − 2 × log-likelihood according to a Chi-squared statistic for additional degrees of freedom added.

## Results

### Descriptive data

**Fig. 1:**
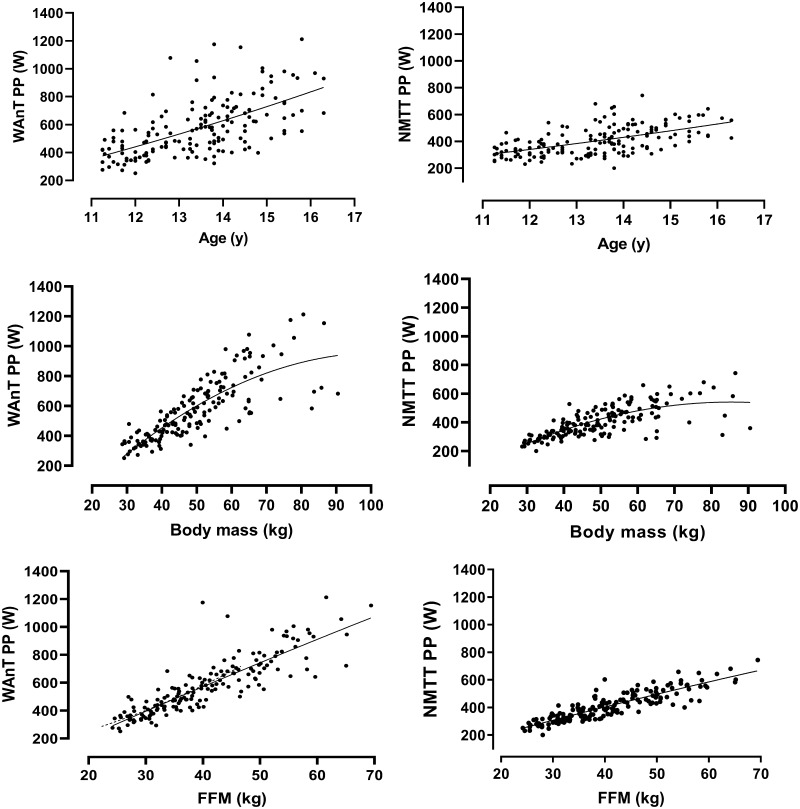
Ergometer-specific peak power output in relation to age, body mass, and estimated fat-free mass in boys. Data are from 163 Wingate anaerobic test (WAnT) determinations and 163 non-motorized treadmill test (NMTT) determinations of 11- to 16-year-old boys’ peak power output. Fat-free mass is estimated from youth-specific equations (Slaughter et al. 1988)

**Fig. 2:**
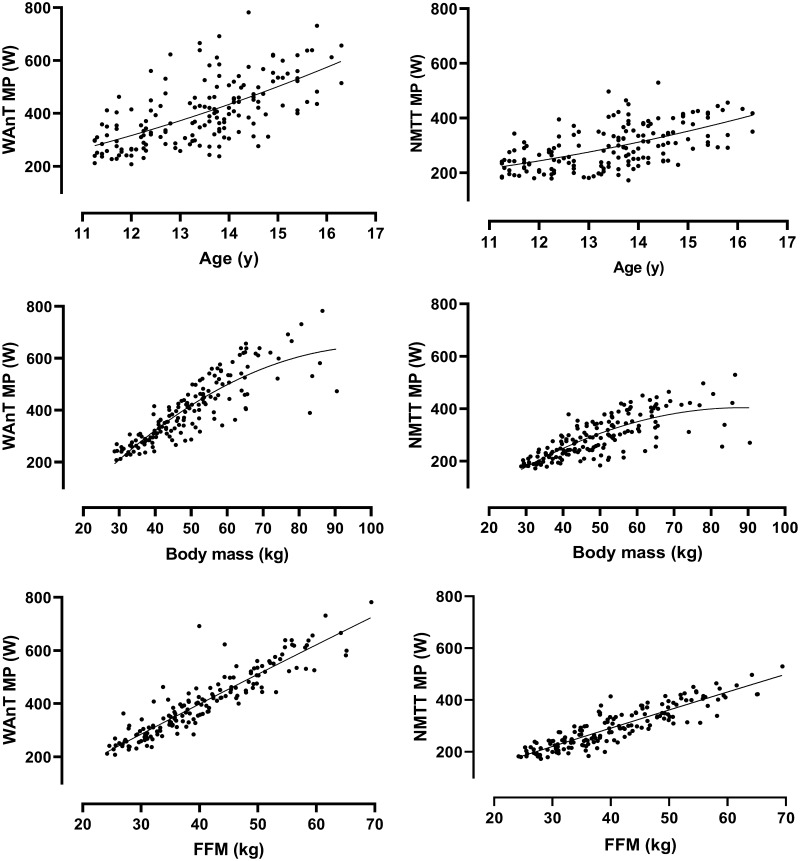
Ergometer-specific mean power output in relation to age, body mass, and estimated fat-free mass in boys. Data are from 163 Wingate anaerobic test (WAnT) determinations and 163 non-motorized treadmill test (NMTT) determinations of 11- to 16-year-old boys’ mean power output. Fat-free mass is estimated from youth-specific equations (Slaughter et al. 1988)

**Fig. 3:**
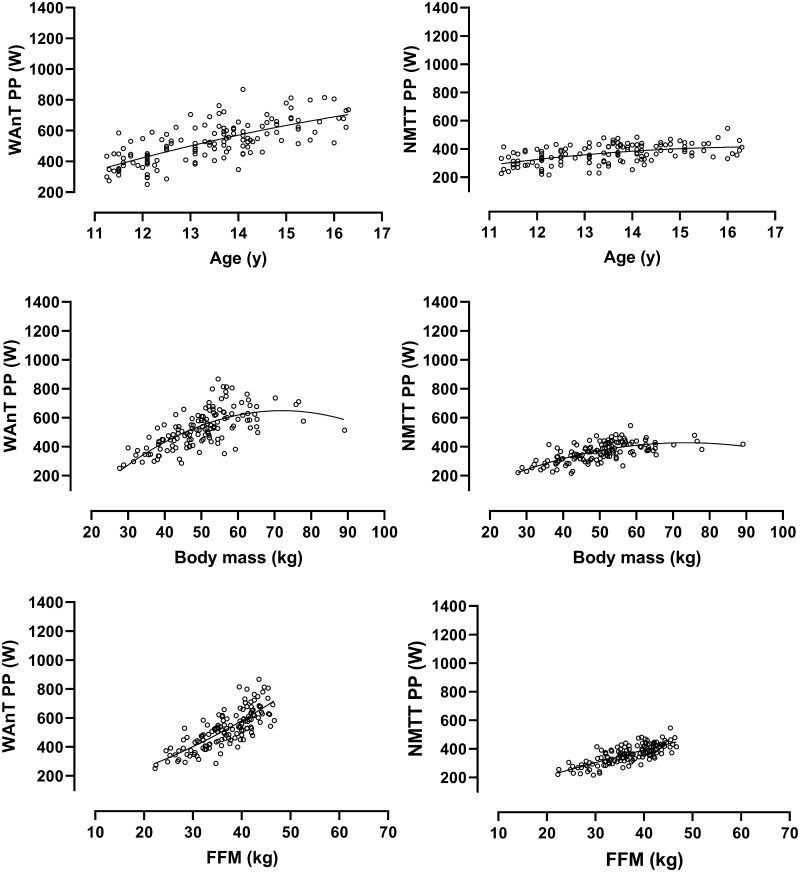
Ergometer-specific peak power output in relation to age, body mass, and estimated fat-free mass in girls. Data are from 138 Wingate anaerobic test (WAnT) determinations and 138 non-motorized treadmill test (NMTT) determinations of 11- to 16-year-old girls’ peak power output. Fat-free mass is estimated from youth-specific equations (Slaughter et al. 1988)

**Fig. 4:**
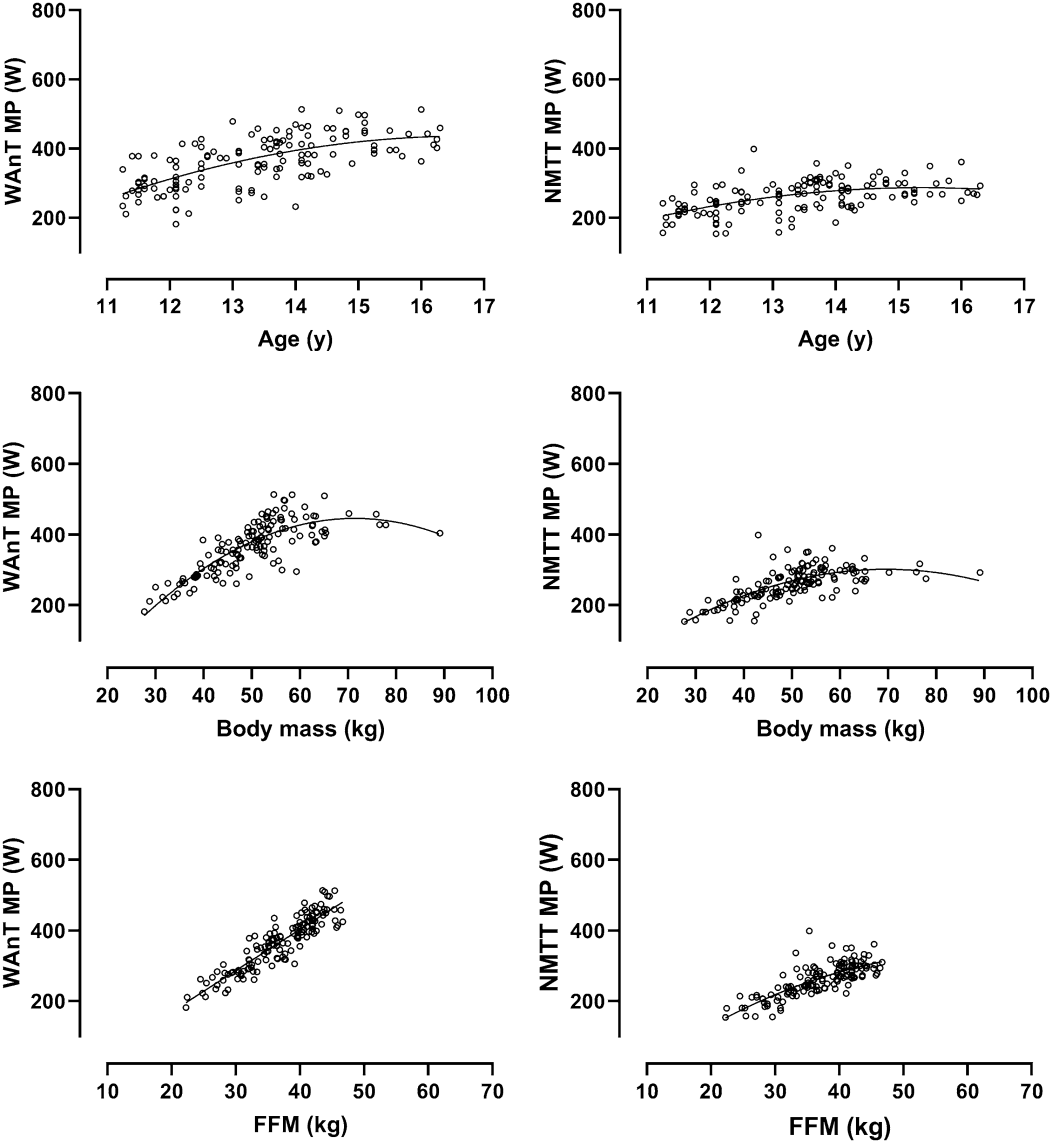
Ergometer-specific mean power output in relation to age, body mass, and estimated fat-free mass in girls. Data are from 138 Wingate anaerobic test (WAnT) determinations and 138 non-motorized treadmill test (NMTT) determinations of 11- to 16-year-old girls’ mean power output. Fat-free mass is estimated from youth-specific equations (Slaughter et al. 1988)

PP and MP on each ergometer by sex in relation to age, body mass, and estimated FFM are illustrated in Figs. 1, 2, 3 and 4. Correlations of PP and MP by sex and ergometer with age (boys, *r* = 0.56–0.64; girls, *r* = 0.50–0.71), body mass (boys, *r* = 0.72–0.85; girls, *r* = 0.63–0.75) and estimated FFM (boys, *r* = 0.86–0.92; girls, *r* = 0.77–0.90) were significant (*p* < 0.05). Correlations between peak $\dot{V}{\text{O}}_{2}$ and age, body mass, and estimated FFM were similar to those of STP with the same variables and are reported in detail elsewhere (Armstrong and Welsman 2019c).

### Multiplicative allometric models

In contrast to traditional methods that require a complete longitudinal data set, both the number of observations per individual and the temporal spacing of the observations may vary within a multilevel analysis. This enables data across 5 years to be collected over a 3-year period. In the present study, there were no significant differences (*p* > 0.05) between those who were unable to attend a test occasion and the rest of their sex-specific group in body mass, skinfold thicknesses, or STP. The models were founded on 301 (138 from girls) measurements of PP and MP on each ergometer supported by age, body mass, skinfold thicknesses, estimated FFM and maturity status and where effects are included in models they are significant (*p* < 0.05).

#### Multiplicative allometric models for PP and MP in the whole data set

The results of modelling the whole data set of PP and MP values from the WAnTs and NMTTs with age and body mass are described in Table 1. In models 1.1 and 1.3, with body mass controlled for, additional positive age effects on both WAnT- and NMTT-determined PP were observed with negative sex terms. Models 1.2 and 1.4 show that, with body mass controlled for, additional positive age effects on both WAnT- and NMTT-determined MP were observed with significant, negative sex and age by sex interaction terms. The age^2^ term was non-significant in all models.

**Table 1:** Ergometer-specific peak power and mean power output in 11- to 16-year olds

	Model 1.1	Model 1.2	Model 1.3	Model 1.4
Response	Log_e_ WAnT PP	Log_e_ WAnT MP	Log_e_ NMTT PP	Log_e_ NMTT MP
Fixed part				
Constant	3.113 (0.230)	2.813 (0.173)	3.661 (0.218)	3.222 (0.218)
Log_e_ body mass	0.823 (0.059)	0.811 (0.044)	0.598 (0.056)	0.628 (0.056)
Age^a^	0.085 (0.009)	0.111 (0.016)	0.034 (0.008)	0.059 (0.019)
Age^2^	ns	ns	ns	ns
Sex	− 0.087 (0.025)	− 0.094 (0.019)	− 0.105 (0.025)	− 0.117 (0.024)
Age by sex	ns	− 0.040 (0.009)	ns	− 0.023 (0.011)
Random part				
Level 2:				
Constant	0.015 (0.003)	0.010 (0.002)	0.017 (0.002)	0.017 (0.002)
Level 1:				
Constant	0.011 (0.001)	0.004 (0.000)	0.006 (0.001)	0.006 (0.001)
Units: level 2	135	135	135	135
Units: level 1	301	301	301	301
− 2 × log-likelihood	− 318.951	− 546.373	− 416.997	− 423.665

Values are model estimates (standard error). Each model founded on 301 determinations of PP or MP

*WAnT* Wingate anaerobic test, *NMTT* non-motorized treadmill test, *PP* peak power output, *MP* mean power output, *ns* not significant (*p* > 0.05)

^a^Age centred on mean age 13.4 years

#### Multiplicative allometric models for PP and MP in boys

Multilevel models for PP for boys are summarized in Table 2. With body mass controlled for, additional positive effects of age on both WAnT- and NMTT-determined PP were identified. Models 2.2 and 2.5 include the additional covariate of sum of triceps and subscapular skinfold thicknesses providing, with body mass, a surrogate for FFM. Having controlled for body mass and skinfold thicknesses, a positive effect of age was observed in model 2.2 although this was considerably reduced compared to model 2.1. In model 2.5, the age effect was not significant. With the introduction of skinfolds, models 2.2 and 2.5 were better statistical fits for the data than model 2.1 and model 2.4, respectively. Similarly, the introduction of estimated FFM, as in models 2.3 and 2.6 produced better fit models than models 2.1 and 2.4, respectively. Model 2.2 was a better statistical fit than model 2.3, whereas model 2.6 was a better fit than model 2.5.

**Table 2:** Ergometer-specific peak power output in 11- to 16-year-old boys

	Model 2.1	Model 2.2	Model 2.3	Model 2.4	Model 2.5	Model 2.6
Response	Log_e_ WAnT PP	Log_e_ WAnT PP	Log_e_ WAnT PP	Log_e_ NMTT PP	Log_e_ NMTT PP	Log_e_ NMTT PP
Fixed part						
Constant	2.562 (0.318)	2.221 (0.280)	2.201 (0.295)	3.458 (0.313)	3.249 (0.183)	2.825 (0.152)
Log_e_ body mass	0.965 (0.082)	1.243 (0.084)	–	0.650 (0.081)	0.894 (0.047)	–
Age^a^	0.078 (0.013)	0.032 (0.014)	0.033 (0.014)	0.034 (0.012)	ns	ns
Age^2^	ns	ns	ns	ns	ns	ns
Log_e_ skinfolds	–	− 0.250 (0.040)	–	–	− 0.250 (0.030)	–
Log_e_ FFM	–	–	1.117 (0.080)	–	–	0.858 (0.041)
Random part						
Level 2:						
Constant	0.020 (0.004)	0.014 (0.003)	0.015 (0.003)	0.022 (0.004)	0.011 (0.002)	0.008 (0.002)
Level 1:						
Constant	0.009 (0.001)	0.008 (0.001)	0.009 (0.001)	0.007 (0.001)	0.007 (0.001)	0.006 (0.001)
Units: level 2	72	72	72	72	72	72
Units: level 1	163	163	163	163	163	163
− 2 × log-likelihood	− 174.521	− 209.559	− 194.610	− 204.123	− 246.081	− 261.716

Values are model estimates (standard error). Each model founded on 163 determinations of PP

*FFM* fat-free mass estimated from youth-specific equations (Slaughter et al. 1988), *WAnT* Wingate anaerobic test, *NMTT* non-motorized treadmill test, *PP* peak power output, *ns* not significant (*p* > 0.05), – not entered

^a^Age centred on mean age 13.4 years

Multilevel models for MP for boys are summarized in Table 3. In model 3.1 and model 3.4, with body mass controlled for, additional positive effects of age were observed. The entry of sum of skinfolds in models 3.2 and 3.5 yielded negative terms and enlarged positive contributions from body mass. In model 3.2, an additional positive but reduced effect of age on MP was noted. In model 3.5, the age effect was not significant. Models 3.2 and 3.5 offer better statistical fits for the data than model 3.1 and model 3.4. The introduction of estimated FFM, as in models 3.3 and 3.6, produced models for MP which were better statistical fits than models 3.1 and 3.4, respectively. Model 3.2 was a better fit than model 3.3. The difference in fit between models 3.5 and 3.6 was not significant. The effects of maturity status were investigated but did not contribute significantly to any of the boys’ PP or MP models.

**Table 3:** Ergometer-specific mean power output in 11- to 16-year old boys

	Model 3.1	Model 3.2	Model 3.3	Model 3.4	Model 3.5	Model 3.6
Response	Log_e_ WAnT MP	Log_e_ WAnT MP	Log_e_ WAnT MP	Log_e_ NMTT MP	Log_e_ NMTT MP	Log_e_ NMTT MP
Fixed part						
Constant	2.541 (0.234)	2.255 (0.194)	2.319 (0.210)	3.149 (0.304)	2.964 (0.178)	2.666 (0.147)
Log_e_ body mass	0.881 (0.060)	1.118 (0.057)	–	0.647 (0.078)	0.866 (0.044)	–
Age^a^	0.063 (0.009)	0.025 (0.009)	0.027 (0.010)	0.031 (0.012)	ns	ns
Age^2^	ns	ns	ns	ns	ns	ns
Log_e_ skinfolds	–	− 0.215 (0.026)	–	–	− 0.225 (0.029)	–
Log_e_ FFM	–	–	0.990 (0.057)	–	–	0.814 (0.040)
Random part						
Level 2:						
Constant	0.012 (0.002)	0.007 (0.002)	0.009 (0.002)	0.023 (0.004)	0.013 (0.003)	0.011 (0.002)
Level 1:						
Constant	0.004 (0.001)	0.003 (0.000)	0.003 (0.001)	0.005 (0.001)	0.005 (0.001)	0.005 (0.001)
Units: level 2	72	72	72	72	72	72
Units: level 1	163	163	163	163	163	163
− 2 × log-likelihood	− 296.951	− 352.013	− 326.420	− 234.349	− 273.151	− 272.754

Values are model estimates (standard error). Each model founded on 163 determinations of MP

*FFM* fat-free mass estimated from youth-specific equations (Slaughter et al. 1988), *WAnT* Wingate anaerobic test, *NMTT* non-motorized treadmill test, *MP* mean power output, *ns* not significant (*p* > 0.05), – not entered

^a^Age centred on mean age 13.4 years

#### Multiplicative allometric models for PP and MP in girls

Multilevel models for PP for girls are summarized in Table 4. In models 4.1 and 4.4, with body mass controlled for, additional positive effects of age were identified. With the entry of skinfold thicknesses in models 4.2 and 4.5, a positive effect of age was observed in model 4.2 but in model 4.5 the age effect was not significant. Models 4.2 and 4.5 were better statistical fits for the data than models 4.1 and 4.4, respectively. The introduction of estimated FFM in models 4.3 and 4.6 produced models for PP which were better statistical fits than models 4.1 and 4.4, respectively. Model 4.5 was a better fit than model 4.6.

**Table 4:** Ergometer-specific peak power output in 11- to 16-year-old girls

	Model 4.1	Model 4.2	Model 4.3	Model 4.4	Model 4.5	Model 4.6
Response	Log_e_ WAnT PP	Log_e_ WAnT PP	Log_e_ WAnT PP	Log_e_ NMTT PP	Log_e_ NMTT PP	Log_e_ NMTT PP
Fixed part						
Constant	3.898 (0.322)	3.449 (0.329)	2.883 (0.405)	3.916 (0.299)	3.223 (0.203)	2.693 (0.228)
Log_e_ body mass	0.599 (0.083)	0.863 (0.110)	–	0.505 (0.077)	0.881 (0.062)	–
Age^a^	0.097 (0.012)	0.074 (0.013)	0.068 (0.014)	0.035 (0.010)	ns	ns
Age^2^	ns	ns	ns	ns	ns	ns
Log_e_ skinfolds	–	− 0.179 (0.053)	–	–	− 0.238 (0.039)	–
Log_e_ FFM	–	–	0.932 (0.113)	–	–	0.888 (0.063)
Random part						
Level 2:						
Constant	0.009 (0.003)	0.007 (0.002)	0.007 (0.002)	0.012 (0.003)	0.008 (0.002)	0.008 (0.002)
Level 1:						
Constant	0.012 (0.002)	0.012 (0.002)	0.013 (0.002)	0.005 (0.001)	0.005 (0.001)	0.005 (0.001)
Units: level 2	63	63	63	63	63	63
Units: level 1	138	138	138	138	138	138
− 2 × log-likelihood	− 158.994	− 169.537	− 168.090	− 221.778	− 242.604	− 237.907

Values are model estimates (standard error). Each model founded on 138 determinations of PP

*FFM* fat-free mass estimated from youth-specific equations (Slaughter et al. 1988), *WAnT* Wingate anaerobic test, *NMTT* non-motorized treadmill test, *PP* peak power output, *ns* not significant (*p* > 0.05), – not entered

^a^Age centred on mean age 13.4 years

Multilevel models for MP for girls are described in Table 5. In models 5.1 and 5.4 with body mass controlled for, additional positive effects of age with additional small age^2^ terms were observed. The entry of skinfolds into models 5.2 and 5.5 offered better statistical fits for the data than models 5.1 and 5.4, respectively. Models 5.3 and 5.5 were the models with the best statistical fit for WAnT- and NMTT-determined MP, respectively. The effects of maturity status were investigated but did not contribute significantly to any of the girls’ PP or MP models.

**Table 5:** Ergometer-specific mean power output in 11- to 16-year-old girls

	Model 5.1	Model 5.2	Model 5.3	Model 5.4	Model 5.5	Model 5.6
Response	Log_e_ WAnT MP	Log_e_ WAnT MP	Log_e_ WAnT MP	Log_e_ NMTT MP	Log_e_ NMTT MP	Log_e_ NMTT MP
Fixed part						
Constant	3.323 (0.255)	2.300 (0.159)	1.619 (0.175)	3.420 (0.285)	2.772 (0.182)	2.312 (0.204)
Log_e_ body mass	0.659 (0.065)	1.159 (0.049)	–	0.550 (0.073)	0.881 (0.055)	–
Age^a^	0.048 (0.009)	ns	ns	0.028 (0.009)	ns	ns
Age^2^	− 0.008 (0.003)	ns	ns	− 0.008 (0.003)	ns	ns
Log_e_ skinfolds	–	− 0.290 (0.029)	–	–	− 0.202 (0.035)	–
Log_e_ FFM	–	–	1.186 (0.049)	–	–	0.901 (0.057)
Random part						
Level 2:						
Constant	0.007 (0.002)	0.003 (0.001)	0.003 (0.001)	0.011 (0.002)	0.008 (0.002)	0.008 (0.002)
Level 1:						
Constant	0.005 (0.001)	0.004 (0.001)	0.004 (0.001)	0.004 (0.001)	0.004 (0.001)	0.004 (0.001)
Units: level 2	63	63	63	63	63	63
Units: level 1	138	138	138	138	138	138
− 2 × log-likelihood	− 262.180	− 298.932	− 303.731	− 258.292	− 274.441	− 268.632

Values are model estimates (standard error). Each model founded on 138 determinations of MP

*FFM* fat-free mass estimated from youth-specific equations (Slaughter et al. 1988), *WAnT* Wingate anaerobic test, *NMTT* non-motorized treadmill test, *MP* mean power output, *ns* not significant (*p* > 0.05), – not entered

^a^Age centred on mean age 13.4 years

#### Multiplicative allometric models for MP including peak $\dot{V}{\text{O}}_{2}$

The models in Table 6 illustrate the entry of ergometer-specific peak $\dot{V}{\text{O}}_{2}$ s into the models with best statistical fit for MP in boys (WAnT: model 3.2; NMTT: model 3.5) and girls (WAnT: model 5.3). Although model 5.5 provided the best statistical fit for NMTT MP in girls, once treadmill peak $\dot{V}{\text{O}}_{2}$ was added as an additional covariate, the model based on estimated FFM (model 5.6) rather than mass and skinfold thicknesses (model 5.5) provided the best statistical fit. In models 6.1 and 6.2 (boys), peak $\dot{V}{\text{O}}_{2}$ made a significant positive contribution to explaining MP in addition to the contributions of body mass and skinfold thicknesses and, in the case of WAnT–determined MP, age. In models 6.3 and 6.4 (girls), peak $\dot{V}{\text{O}}_{2}$ made a significant positive contribution to explaining MP in addition to the contribution of estimated FFM. In both sexes on both ergometers, the models including peak $\dot{V}{\text{O}}_{2}$ provided the best statistical fits for MP.

**Table 6:** Ergometer-specific mean power output including peak oxygen uptake in 11- to 16-year olds

	Boys		Girls	
	Model 6.1	Model 6.2	Model 6.3	Model 6.4
Response	Log_e_ WAnT MP	Log_e_ NMTT MP	Log_e_ WAnT MP	Log_e_ NMTT MP
Fixed part				
Constant	2.869 (0.250)	3.735 (0.262)	2.289 (0.230)	3.014 (0.276)
Log_e_ body mass	0.855 (0.091)	0.533 (0.098)	–	–
Age^a^	0.027 (0.009)	ns	ns	ns
Age^2^	ns	ns	ns	ns
Log_e_ skinfolds	− 0.153 (0.030)	− 0.166 (0.033)	–	–
Log_e_ FFM	–		0.944 (0.074)	0.630 (0.093)
Log_e_ CE $\dot{V}{\text{O}}_{2}$	0.278 (0.077)	–	0.299 (0.072)	–
Log_e_ TM $\dot{V}{\text{O}}_{2}$	–	0.366 (0.090)	–	0.340 (0.094)
Random part				
Level 2:				
Constant	0.006 (0.001)	0.009 (0.002)	0.002 (0.001)	0.007 (0.002)
Level 1:				
Constant	0.003 (0.000)	0.005 (0.001)	0.004 (0.001)	0.004 (0.001)
Units: level 2	72	72	63	63
Units: level 1	163	163	138	138
− 2 × log-likelihood	− 364.075	− 286.760	− 319.327	− 280.865

Values are model estimates (standard error). Models 6.1 and 6.2 founded on 163 determinations of MP, age, body mass, skinfold thicknesses and peak oxygen uptake. Models 6.3 and 6.4 founded on 138 determinations of MP, estimated FFM and peak oxygen uptake

*FFM* fat-free mass estimated from youth-specific equations (Slaughter et al. 1988), *WAnT* Wingate anaerobic test, *NMTT* non-motorized treadmill test, *MP* mean power output, *CE*$\dot{V}O_{2}$ cycle ergometer-determined peak oxygen uptake, *TM*$\dot{V}O_{2}$ treadmill-determined peak oxygen uptake, *ns* not significant (*p* > 0.05), – not entered

^a^Age centred on mean age 13.4 years

## Discussion

Descriptive data focusing on STP in relation to age or a single morphological variable provide limited insights but are briefly outlined herein for cross-study comparative purposes. Ergometer-specific data describing PP and MP in relation to age, body mass, and estimated FFM, respectively, are shown in Figs. 1, 2, 3 and 4. The descriptive data and correlations with age, body mass, and estimated FFM from the WAnT are in accord with those reported in cross-sectional studies (Van Praagh and Doré 2002; Williams and Ratel 2017) but there are no corresponding comparative data from NMTTs. All reported correlations are significant but the strongest correlations in both sexes are between STP and estimated FFM. On both ergometers and in both sexes, the relationships of PP and MP with age and estimated FFM are near-linear although it is notable that there are wider individual variations in WAnT-determined data, particularly in relation to age. In all cases, PP and MP increase with body mass with a tendency to begin levelling-off at ~ 60 kg in girls and ~ 70 kg in boys.

The baseline multiplicative allometric models 1.1–1.4 presented in Table 1 are each founded on 301 determinations of PP or MP and illustrate a similar picture of the development of STP. The positive age and negative sex terms in models 1.1 and 1.3 show that with body mass controlled for both WAnT- and NMTT-determined PP increases with age with the age effect smaller in girls. In models 1.2 and 1.4 describing the development of MP, an additional negative age by sex interaction demonstrates that on both ergometers with body mass controlled for MP increases with age at a greater rate in boys. Girls normally enter puberty before similarly aged boys (Malina 2017) and at the onset of the present study 56% and 23% of 11-year-old boys and girls, respectively, were pre-pubertal (i.e. at PH stage 1). The size of maturity status-driven changes in morphological and physiological variables related to performance is sex specific and it is, therefore, appropriate to analyse the development of boys’ and girls’ STP in sex-specific models rather than combining data as in Table 1.

The patterns of development of PP and MP are remarkably similar in boys and girls regardless of ergometer but the relative size of contribution of the morphological explanatory variables is sex specific. The baseline models (models 2.1, 3.1, 4.1, and 5.1) show that, with body mass controlled for, age exerts a significant additional effect on PP and MP in both sexes. The entry of maturity status to the STP baseline models had no additional effect on either PP or MP. This is in accord with the development of aerobic power in the same girls but in contrast with data from the same boys where PH stages 2–5 each made a significant positive contribution to explaining the development of peak $\dot{V}{\text{O}}_{2}$ (Armstrong and Welsman 2019c).

The introduction of sum of skinfolds into models 2.2, 2.5, 3.2, 3.5, 4.2, 4.5, 5.2, and 5.5 resulted in significant negative exponents for skinfolds and increased body mass exponents. The marked increase in body mass exponents was also observed in corresponding models of peak $\dot{V}{\text{O}}_{2}$ (Armstrong and Welsman 2019c) and it has been attributed to the effect that excess fat mass has on increasing body mass without an increase in the exercise performance variable (Vanderburgh et al. 1995). With the exception of the girls’ MP model (model 5.2) where the contribution of age was not significant, reduced but significant age terms were present in WAnT-determined STP models. In all NMTT-determined STP models, the age term was not significant. In all cases, the entry of skinfold thicknesses, which act with body mass as a surrogate for FFM, resulted in models with a significantly better statistical fit than those founded on age and body mass. Models 2.3, 2.6. 3.3, 3.6, 4.3, 4.6, 5.3, and 5.6 founded on FFM estimated from the youth-specific equations reported by Slaughter et al. (1988) reflect those of skinfold thicknesses plus body mass confirming the strong influence of FFM on PP and MP. The models including estimates of FFM are in accord with those describing the development of peak $\dot{V}{\text{O}}_{2}$ in the same participants where additional effects of maturity status in boys were negated by the introduction to the baseline model of skinfold thicknesses or the replacement of body mass with estimated FFM (Armstrong and Welsman 2019c).

The multiplicative allometric models illustrate the powerful influence of FFM on both PP and MP. The influence of sex-specific maturity status on FFM is evidenced by reported changes in FFM being at their zenith around the time of peak height velocity (PHV). Boys’ FFM increases by ~ 83% over the period 2 years pre-PHV to 2 years post-PHV with girls’ FFM increasing ~ 31% over a 2-year period centred on PHV (Armstrong 2019; Baxter-Jones et al. 2003).

In both cycling and running, STP is primarily developed from the leg muscles and sex differences have generally been attributed to boys’ greater leg or thigh muscle volume (De Ste Croix et al. 2001: Santos et al. 2003; Welsman et al. 1997). On the other hand, it has been argued that as cycling and particularly running involve other muscles (e.g. trunk and arm muscles) FFM may be the most influential morphological variable in the development of STP (Doré et al. 2000, 2001). Certainly with the experimental challenges and costs of determining leg or thigh muscle volumes (Malina et al. 2004; Santos et al. 2003; Winsley et al. 2003), FFM estimated either from body mass and skinfolds directly or calculated from youth-specific equations presents a pragmatic morphological variable with which to scale and compare STP in youth.

FFM is not the only maturity status-driven variable contributing to the development of PP and MP. Other factors intrinsic to muscle but not investigated herein include changes in muscle structure, muscle fibre type, muscle activation (i.e. the decline in activation deficit), and muscle metabolism. These factors have been reviewed elsewhere in relation to the ethical and methodological challenges of exploring their effects on exercise performance but empirical evidence is sparse (Armstrong et al. 2017; Barker and Armstrong 2010; Dotan et al. 2012; Malina et al. 2004).

The morphological variables influencing the development of MP are similar to those describing PP but, unlike PP, MP represents an interplay of anaerobic and aerobic metabolism. MP is an important component of performance in many youth sports (Armstrong 2019) but few studies have investigated the contribution of $\dot{V}{\text{O}}_{2}$ to MP. It has been demonstrated empirically that the pulmonary $\dot{V}{\text{O}}_{2}$ kinetics response at the beginning of high intensity exercise slows from 11 to 16 years (Breese et al. 2010; Fawkner and Armstrong 2004) so the anaerobic contribution to MP would be expected to increase with age. The net $\dot{V}{\text{O}}_{2}$ during a 30 s WAnT has been reported to be 45% higher in 10-year-old boys than 22-year-old men (Hebestreit et al. 1993). It has been suggested that the aerobic contribution to cycling MP lies within the range 16–45% in 9- to 12-year olds (Chia 2006; Chia et al. 1997) but variations in the mechanical efficiency of cycling with age make it difficult to be more precise. Chia (2006) estimated that 10- to 12-year-old boys and girls attain 67% and 73% of peak $\dot{V}{\text{O}}_{2}$, respectively, during WAnTs. Phase 2 pulmonary $\dot{V}{\text{O}}_{2}$ kinetics time constants from 11- to 16-year olds reported from a number of sources (tabulated by Barker and Armstrong 2017) lend support to these estimates by indicating that values of ~ 70–75% of peak $\dot{V}{\text{O}}_{2}$ are likely to be approached within 30 s of high intensity cycling or running.

As illustrated in Table 6, when ergometer-specific peak $\dot{V}{\text{O}}_{2}$ was entered it made a significant contribution additional to that of either body mass and skinfold thicknesses (as a surrogate of FFM) or estimated FFM in explaining the development of MP in both sexes, on both ergometers. The final models (models 6.1–6.4), including peak $\dot{V}{\text{O}}_{2}$ and either sum of skinfold thicknesses and body mass (boys) or estimated FFM (girls) and in the case of boys’ WAnT-determined MP age, present the best statistical fit models of the development of MP. The influence of peak $\dot{V}{\text{O}}_{2}$ on cycling and running MP had not previously been investigated in youth but it is clear that it makes a significant contribution to the development of MP in both sexes, on both ergometers.

### Strengths and limitations

Potential limitations to this study lie in the tests themselves. The well-established WAnT uses apparatus readily available in most paediatric exercise laboratories but to obtain true maximal power output on a cycle ergometer the braking force should be matched to muscle capability so that the test can be performed at optimal pedal cadence. In a longitudinal study, this would ideally require the optimal braking force to be determined specifically for each of PP and MP on each test occasion. However, as mentioned earlier, it appears that in the present age group the WAnT is generally resilient to moderate variations in body mass-related braking force around the conventional value of 0.74 N kg^−1^ and therefore appropriate for large-scale studies (Dotan and Bar-Or 1983; Santos et al. 2002). Nevertheless, having established in this study the dominant influence of FFM a braking force based on FFM is likely to be superior to one based on body mass and this should perhaps be prioritized in future studies using the WAnT. Ideally FFM would be directly determined on each test occasion rather than estimated from body mass and skinfold thicknesses but this is not currently feasible in studies involving several hundred assessments. Moreover, measures of body fat of the same young people have recently been shown to vary widely across established laboratory techniques (Ferri-Morales et al. 2018).

The NMTT station was purpose built to assess the performance of children and adolescents and some off-the-shelf purchased components were evaluated and rejected in favour of self-developed components. For example, the tether supplied with the NMT was highly extensible and replaced during development with a belt which was more rigid but did not cause discomfort to the young runner. The work done sprinting on an NMT can be resolved into vertical and horizontal components and herein it is the horizontal component, the component which maintains treadmill motion, which is measured. In the development of the NMTT station, vertical displacement of the belt during a full gait cycle (heel strike to heel strike of the same foot) was evaluated by digitized video analysis. Vertical displacement did not exceed 5 cm throughout the gait cycle and the resultant error in the measurement of power output was reported as being consistent at less than 2.5%, in both boys and girls, and considered to be insignificant with regard to evaluating overall performance (Sutton et al. 2000). In brief, the development and evaluation of an appropriate NMTT station and the subsequent habituation of children and adolescents to running maximally on an NMT are resource consuming but once established the NMTT station provides a valuable laboratory tool for a range of paediatric exercise investigations as alluded to earlier in this paper (e.g. Oliver et al. 2006, 2007; Ratel et al. 2004, 2006).

The principal strength of the study lies in the provision of new insights into the development of STP in 11- to 16-year olds. It is the first investigation to use an NMTT in a longitudinal study of children and adolescents and the first to use both cycling and running tests to determine PP and MP. The use of both cycling and running exercise modes has enabled ergometer-specific peak $\dot{V}{\text{O}}_{2}$ to be incorporated into analyses of MP and to demonstrate the influence of peak $\dot{V}{\text{O}}_{2}$ in explaining the development of cycling and running MP, even when morphological variables have been controlled for. A major and unique strength of the study lies in the adoption of a multiplicative allometric modelling approach. This allowed the effects of age, body mass, FFM, and maturity status to be partitioned concurrently within an allometric framework to provide a sensitive, sex-specific interpretation of the development of STP. In addition, collectively the present study and a parallel investigation (Armstrong and Welsman 2019c) facilitate a direct comparison of the development of the STP and aerobic power of the same 11- to 16-year olds.

## Conclusions

The data demonstrate that, (1) in accord with the development of peak $\dot{V}{\text{O}}_{2}$, maturity status-driven FFM is the most powerful morphological influence on the development of PP and MP in 11- to 16-year olds, on both ergometers in both sexes; (2) with FFM controlled for peak $\dot{V}{\text{O}}_{2}$ makes an additional contribution to explaining the development of MP on both ergometers in both sexes; (3) an NMTT provides an appropriate model for investigating running STP in youth and offers a complementary test to the established WAnT; and (4) the development of STP is sex specific but within sex multiplicative allometric models of running- and cycling-determined PP and MP were similar, suggesting that either mode of exercise can be used in future studies of STP in youth.

## Abbreviations

FFM: Fat-free mass
MP: Mean power output
NMT: Non-motorized treadmill
OPP: Optimized peak power output
$$ \dot{V}{\text{O}}_{2} $$: Oxygen uptake
PHV: Peak height velocity
PP: Peak power output
PH: Pubic hair
STP: Short-term power output
WAnT: Wingate anaerobic test
